# The association between plain water intake and periodontitis in the population aged over 45: a cross-sectional study based on NHANES 2009–2014

**DOI:** 10.1186/s12903-023-03809-y

**Published:** 2024-01-05

**Authors:** Xianxian Li, Lili Wang, Luming Yang, Xianghong Liu, Henglang Liu, Yandong Mu

**Affiliations:** 1 Stomatology Department, Sichuan Provincial People’s Hospital, University of Electronic Science and Technology of China, Chengdu, 610072 China; 2 School of Stomatology, Southwest Medical University, Luzhou, 646699 China

**Keywords:** Plain water intake, Periodontitis, Cross-sectional study, Population-based study, NHANES

## Abstract

**Background:**

Numerous studies have demonstrated the impact of beverage consumption on overall health and oral health. Specifically, high consumption of sugar-sweetened beverages and coffee has been associated with an increased risk of metabolic disorders and periodontitis. Conversely, high intake of plain water has been linked to various health benefits, including weight management and reduced energy intake. However, no previous studies have explored the potential association between plain water intake and the risk of periodontitis.

**Objectives:**

Our objective was to investigate the relationship between plain water consumption and periodontitis in a middle-aged and elderly population.

**Methods:**

The present cross-sectional study was conducted among participants aged ≥ 45 in the 2009–2014 National Health and Nutrition Examination Surveys. Multivariable regression analysis, subgroup analysis and smooth fitting tests were conducted to explore the independent relationship between plain water intake and periodontitis.

**Results:**

A total of 5,882 participants were enrolled,62.02% have periodontitis. Periodontitis patients have lower plain water intake. The multivariable regression tests showed that the risk of periodontitis decreased with increased plain water intake quartiles (Q4 OR = 0.78; 95%CI 0.62–0.96) after fully adjustment. Subgroup analysis and interaction tests showed that gender, age, smoking, diabetes, hypertension or BMI does not significantly interact with the association. However, the relation was significant in males (Q4 OR = 0.64; 95%CI 0.47–0.86) but not in females (Q4 OR = 0.97;95% CI 0.71–1.31). In the smoothed curve fits stratified by gender, the curve for male participants displayed as a U-shape, with an optimal plain water intake at 1200 ml/day. For males drinking plain water less than 1200 ml/day, the risk of periodontitis decreased by 24% with each increase of 500 ml plain water intake (OR = 0.76, 95%CI 0.66–0.87, *p* < 0.001).

**Conclusions:**

Together, the results showed that plain water intake is negatively associated with periodontitis risk in US middle aged and elderly population. Further studies are needed to investigate the mechanism unites this association. Attention should be given to adequate plain water intake when considering dietary suggestions to the population at high risk of developing periodontitis, especially for men.

## Introduction

Periodontitis, a common oral health issue affecting millions worldwide, impacts over half of the male and one-third of the female population above 30 years in the United States [1]. The prevalence of periodontitis tends to increase with age. According to the 2009–2014 National Health and Nutritional Examination Surveys (NHANES), among individuals aged between 45 and 64, the prevalence of periodontitis is approximately 46.0%, whereas in those aged 65 or older, the number increases to 59.8% [1]. Periodontitis is characterized by inflammation and destruction of the periodontal supporting tissues and is the most common reason for tooth loss in adults. Furthermore, numerous studies have demonstrated that periodontitis has negative impacts on systemic diseases such as cardiovascular disease, diabetes, cancer, rheumatoid arthritis, respiratory disease, Alzheimer’s disease, and chronic kidney disease (CKD) [2]. Therefore, preventing and treating periodontitis is crucial for overall health.

In addition to well-known periodontitis-related factors such as poor oral hygiene habits, smoking, and diabetes, recent research has shown that dietary factors are also strongly linked to the likelihood of developing periodontitis [3–6]. Water, the most fundamental and essential nutrient in our diet, is involved in nearly all our physiological functions and is acquired through the liquid in food and beverages. The amount and source of water consumed play crucial roles in oral and systemic health. Dehydration, for example, can lead to major health issues, and drinking sugary beverages has been associated with a higher risk of developing diabetes [7], obesity [8], cancer [9] and dental caries [10]. Alternatively, plain water is recommended as a healthier beverage choice than sugar-sweetened beverages to promote adequate hydration while reducing added sugar intake, according to The Dietary Guidelines for Americans 2015–2020 [11, 12]. In fact, higher plain water intake has been found to reduce the risk of nonalcoholic fatty liver disease [13], obesity and diabetes [7, 12], chronic kidney disease [14], depression, and anxiety [15]. Interestingly, all of these diseases and metabolic disorders are known to exacerbate or be closely associated with periodontitis [16–19]. Therefore, we speculate that higher plain water intake may have an impact on general metabolic conditions and provide benefits in reducing the risk of periodontitis.

However, no study to date has explored the relationship between plain water intake and periodontal disease. Hence, we conducted this study to shed light on the relationship between plain water intake and periodontal disease, with the results potentially contributing to the development of dietary recommendations for individuals at high risk of developing periodontal disease.

## Materials and methods

### Data and sample source

Data used in the present study were obtained from the National Health and Nutrition Examination Surveys for survey years 2009–2010,2011–2012 and 2013–2014. The survey is conducted by the Centers for Disease Control and Prevention’s (CDC) National Center for Health Statistics. A complex stratified, multistage probability cluster sampling design was utilized to recruit a representative sample of the entire US population. By a combination of in-home interviews and standardized physical examinations conducted in mobile examination centers (MEC), NHANES aims to monitor the health and nutritional status of the civilian noninstitutionalized U.S. population. We chose the data from 2009 to 2014 because during these years, participants over the age of 30 underwent a full-mouth periodontal examination. Dietary data were obtained through two separate 24-hour dietary recall interviews. The first dietary recall interview is collected in-person in the Mobile Examination Center (MEC) and the second interview is collected by telephone 3 to 10 days later. More detailed information on the study design, sampling scheme, response rates, and survey protocols, as well as the released publicly available data can be found online at https://www.cdc.gov/nchs/nhanes.

**Fig. 1:**
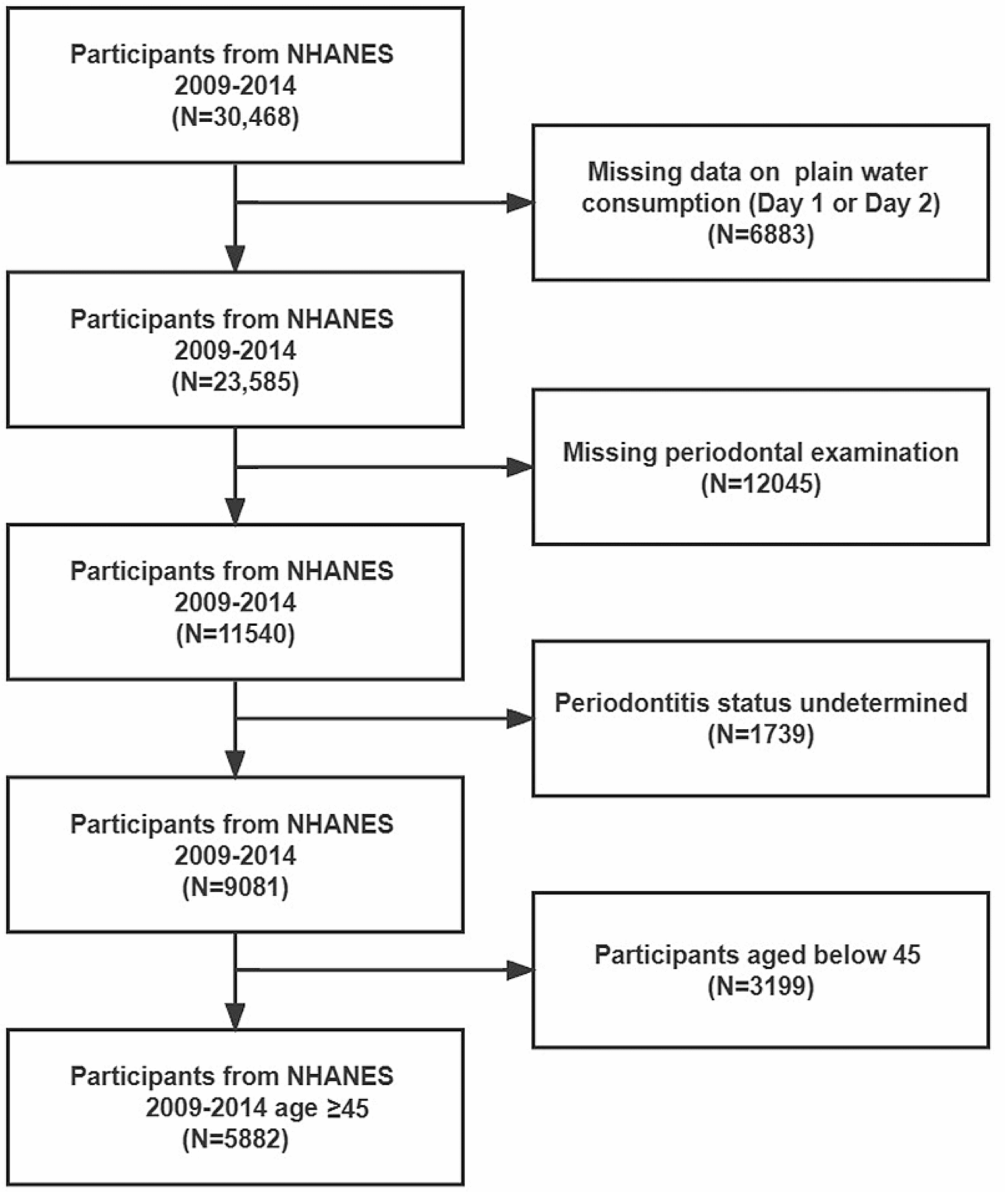
The flow chart of sample selection form NHANES 2009–2014

In this study, NHANES 2009–2014 data were retrieved with the following inclusion criteria: participants who reported the recalled plain water intake on both dietary interviews and received periodontal examination (n = 11,540). 1739 participants were excluded from the study due to edentulism (lack of teeth) or having only one tooth, which made it impossible to determine their periodontal status. This study focused mainly on periodontal status in middle-aged and elderly individuals, so the participants aged below 45 were excluded (n = 3199). The final sample size was n = 5882. (The flow chart was shown in Fig. 1).

### Plain water intake measurements

Total plain water intake (ml/day) was defined as the total volume of water consumed (including plain tap water, water from drinking fountains or water coolers, bottled water, and spring water) over 24 h. Each participant recalled their water intake during the first face-to-face interview and then reported it again during a telephone interview 3–10 days later. The average water intake from the two reports, which was closer to the actual water intake, was used for subsequent analysis.

### Periodontitis status assessments

All teeth except for the third molar were examined by periodontal probing at 6 sites per tooth: mesiobuccal, middle-buccal, distobuccal, mesiolingual, middle-lingual, and distolingual. Probing depth (PD) and attachment loss (AL) were recorded by trained dental examiners. The periodontitis status of each participant was determined according to the Centers for Disease Control and Prevention American Academy of Periodontology (CDC-AAP) standards [11].

- Mild periodontitis: ≥ 2 interproximal sites with AL ≥ 3 mm, and ≥ 2 interproximal sites with PD ≥ 4 mm (not on same tooth) or one site with PD ≥ 5 mm;
- Moderate periodontitis: ≥ 2 interproximal sites with AL ≥ 4 mm (not on the same tooth), or ≥ 2 interproximal sites with PD ≥ 5 mm (not on same tooth);
- Severe periodontitis: ≥ 2 interproximal sites with AL ≥ 6 mm (not on the same tooth) and ≥ 1 interproximal site with PD ≥ 5 mm.

In this study, periodontitis status includes mild, moderate and severe periodontitis. Non- periodontitis was defined as participants having no evidence of mild, moderate or severe periodontitis.

### Covariables

We collected covariates that may affect the association between plain water intake and periodontitis, including main demographic variables, body mass index (BMI), lifestyle, medical history, total dietary intake, etc. Specifically, these covariates include including age(year), gender (male/female), race (Mexican American/Other Hispanic/Non-Hispanic White/Non-Hispanic Black/Other races), education level(Less than high school/High school or GED/Above high school), Ratio of family income to poverty, BMI(kg/m^2^), smoking status(yes/no), alcohol drinking status(yes/no), history of diabetes(yes/no), hypertension(yes/no), cardiovascular disease(yes/no), chronic kidney disease (yes/no), and physical activity time (min/day), and total energy intake(kcal/day). Smoking status was determined by asking participants “Have you smoked at least 100 cigarettes in your entire life?”. Alcohol drinking status was determined by asking “Have you had at least 12 alcohol drinks per year?” History of diabetes, hypertension, and chronic kidney disease is self-reported, by enquiring “Have you ever been told by a doctor or health professional that you have diabetes/ hypertension/failing kidney?” History of cardiovascular diseases include self-reported history of congestive heart failure, coronary heart disease, angina and heart attack. Physical activity time is calculated by adding up the time (min/day) spent on moderate-intensity and vigorous-intensity physical activities during both work and leisure time. BMI was divided into three categories: <25 (normal weight), 25-29.9 (overweight), and ≥ 30 kg/m2 (obese). The details of inquiries and measurements regarding these covariables can be obtained on the NHANES website.

### Statistical analysis

We presented proportions for categorical variables and means and standard deviations (SD) for continuous variables. Categorical variables were tested using chi-square tests and continuous variables were tested using Student’s t-test to assess differences between participants grouped by periodontitis status. Multivariate logistic regression was used to analyze the association between plain water intake and periodontitis in three different models. In Model I, no covariates were adjusted. Model II adjusted for gender, age and race. Model III adjusted for age, gender, race, education level, ratio of family income to poverty, BMI, smoking status, alcoholic status, hypertension, diabetes, chronic kidney disease, cardiovascular disease, physical activity time and total energy intake. The amount of plain water intake was categorized into four groups based on quartiles. We also treated the amount of plain water intake as a continuous variable, with 500 ml as one unit. Both categorized and continuous plain water intake were analyzed in the multivariate logistic regression. Subgroup analysis of the association between plain water intake and periodontitis was conducted with stratified factors including gender(male/female), age(45–65/≧65), smoking status(yes/no), diabetes(yes/no), hypertension(yes/no), and BMI (normal weight/overweight/obese). Additionally, an interaction term was conducted to assess subgroup heterogeneity. To detect potential nonlinearity, we performed a smooth curve fitting for plain water intake (ml/day) and the odds ratio (OR) for periodontitis. If nonlinearity was detected a two-piecewise linear regression model and log-likelihood ratio test was performed. Data were analyzed by R version 4.2.0(http://www.R-project.org, The R Foundation) and EmpowerStats software (www.empowerstats.com; X & Y Solutions, Inc., Boston MA).

The statistical significance level was set as *p* < 0.05.

## Results

### Baseline characteristics of participants

Table 1 shows that a total of 5,882 participants were enrolled, consisting of 2,838 (48.25%) males and 3,044 (51.75%) females. The average age of participants was 60.42 ± 10.40 years. 3648 participants (62.02%) have periodontitis. The median plain water intake was 747 ml/day. Table 1 shows baseline information of participants stratified by periodontal status. Statistically significant differences were witnessed in age, gender, race, education level, ratio of family income to poverty, smoking status, hypertension, diabetes, chronic kidney disease, cardiovascular disease, physical activity time and plain water intake between the periodontal healthy individuals and participants with periodontitis(*p* < 0.05). Compared to the non-periodontitis group, participants with periodontitis have a higher proportion of males, a higher average age, lower levels of education, more impoverished families, a higher proportion of smokers, poorer basic health conditions with a higher incidence of hypertension, cardiovascular disease, chronic kidney disease, and diabetes. They also reported more time spent on physical activities and a lower intake of plain water.

**Table 1:** Baseline characteristics of participants

	Non-periodontitis	Periodontitis		
N	2234	3648	Standardize diff.	*p*-value
**Age**, (year, mean mean ± SD)	58.6 ± 10.2	61.6 ± 10.4	0.3 (0.2, 0.3)	< 0.001
**Gender**, n (%)			0.41 (0.35, 0.46)	< 0.001
Male	802 (35.90)	2036 (55.81)		
Female	1432 (64.10)	1612 (44.19)		
**Race**, n (%)			0.28 (0.23, 0.33)	< 0.001
Mexican American	195 (8.73)	552 (15.13)		
Other Hispanic	215 (9.62)	360 (9.87)		
Non-Hispanic White	1202 (53.80)	1546 (42.38)		
Non-Hispanic Black	412 (18.44)	874 (23.96)		
Other races	210 (9.40)	316 (8.66)		
**Education level**, n (%)			0.44 (0.39, 0.50)	< 0.001
Less than high school	312 (13.97)	1026 (28.13)		
High school or GED	433 (19.3)	860 (23.57)		
Above high school	1486 (66.52)	1756 (48.14)		
**Ratio of family income to poverty**	3.24 ± 1.63	2.52 ± 1.59	0.44 (0.39, 0.50)	< 0.001
**BMI**	29.46 ± 6.45	29.49 ± 6.55	0.00 (-0.05, 0.06)	0.889
**At least 12 alcohol drinks/1 year?**			0.04 (-0.01, 0.09)	0.358
Yes	1541 (70.98)	2543 (72.57)		
NO	626 (28.83)	957 (27.31)		
**Smoked 100 cigarettes in lifetime?**			0.34 (0.29, 0.39)	< 0.001
Yes	808 (36.17)	1918 (52.58)		
No	1426 (63.83)	1727 (47.34)		
**Physical activity time**, n (%)			0.14 (0.07, 0.20)	< 0.001
Q1	441 (29.30)	579 (25.87)		
Q2	603 (40.07)	828 (37.00)		
Q3	461 (30.63)	831 (37.13)		
**Cardiovascular disease**, n (%)			0.18 (0.13, 0.24)	< 0.001
Yes	134 (6.00)	406 (11.13)		
NO	2100 (94.00)	3242 (88.87)		
**Diabetes**, n (%)			0.17 (0.11, 0.22)	< 0.001
Yes	298 (13.34)	699 (19.16)		
No	1860 (83.26)	2815 (77.17)		
**Chronic kidney diseases**			0.10 (0.05, 0.15)	0.001
Yes	48 (2.15)	135 (3.70)		
NO	2178 (97.49)	3507 (96.13)		
**Hypertension**, n (%)			0.09 (0.03, 0.14)	0.007
Yes	1021 (45.70)	1819 (49.86)		
No	1211 (54.21)	1824 (50.00)		
**Total energy intake**, (kcal, mean ± SD)	1934.70 ± 693.96	1961.31 ± 765.46	0.04 (-0.02, 0.09)	0.180
**Plain water intake**, n (%)			0.17 (0.12, 0.22)	< 0.001
Q1 (< 333 ml)	468 (20.95)	993 (27.22)		
Q2 (333–746 ml)	554 (24.80)	923 (25.30)		
Q3 (747–1324 ml)	581 (26.01)	892 (24.45)		
Q4 (> 1324 ml)	631 (28.25)	840 (23.03)		

### The association between plain water intake and periodontitis

As shown in Table 2, compared with participants in the lowest plain water intake quartile(Q1), the risk of periodontitis of participants in the highest plain water intake quartile(Q4) was significantly decreased in the crude model (OR = 0.63;95%CI,0.54–0.73; p for trend < 0.0001), minimal adjusted model (OR = 0.69;95%CI,0.59–0.81; p for trend < 0.0001). The association was still stable after adjusted for all covariables (OR = 0.78; 95%CI 0.63–0.96; p for trend < 0.05), indicating that the risk of periodontitis decreased by 22%. We then analyzed daily plain water intake as a continuous variable, with each unit representing 500 ml. The results showed that after adjusting the main domestic variables, when the daily water intake increased every 500 ml, the risk of periodontitis decreased by 4% (OR = 0.96; 95%CI 0.93–0.99). In the fully adjusted model III, the increased plain water intake was also associated with lower periodontitis risk, but this association did not meet the statistical significance (OR = 0.97; 95%CI 0.94–1.01).

**Table 2:** Relationship between plain water intake and periodontitis

	Model I	Model II	Model III
	OR^1^ (95%CI^2^)	OR (95%CI)	OR (95%CI)
**Plain water intake (quartiles**)			
Q1	Reference	Reference	Reference
Q2	0.79 (0.67, 0.91)	0.75 (0.64, 0.88)	0.84 (0.68, 1.05)
Q3	0.72 (0.62, 0.84)	0.75 (0.64, 0.88)	0.75 (0.63, 0.96)
Q4	0.63 (0.54, 0.73)	0.69 (0.59, 0.81)	0.78 (0.62, 0.96)
*P* for trend	< 0.0001	< 0.0001	0.031
**Plain water intake, (every 500 ml)**	0.93 (0.91, 0.96)	0.96 (0.93, 0.99)	0.97 (0.94, 1.01)

^1^OR: odds ratio

^2^ 95%CI: 95% confidence interval

Model I: Non-adjusted model

Model II: adjusted for gender, age, race

Model III: adjusted for: age, gender, race, education level, family to ratio of family income to poverty, BMI, smoking status, alcoholic status, hypertension, diabetes, CKD, cardiovascular disease, physical activity time, total energy intake

### Subgroup analysis

Table 3 displays the analysis results, showing that across all subgroups, participants in Q4 consistently have lower risks of periodontitis compared to those in Q1 (all OR < 1). However, in some of the subgroups, the association was not statistically significant. Notably, in the gender-stratified analysis, the association between increased water consumption and reduced risk of periodontitis was significant in men, with a 36% reduction in risk in the Q4 group compared to the Q1 group, but this reduction was not significant in women. In the BMI-stratified analysis, there was a significant 35% decrease in the risk of periodontitis in the obese subgroup; however, this decrease was not significant in the normal-weight group. The interaction test showed that gender, age, smoking, diabetes, hypertension or BMI do not significantly interact with the association between plain water intake and periodontitis (all p for interaction > 0.05).

**Table 3:** Effect size of plain water intake on periodontitis in each subgroup

Characteristic				OR^1^ (95% CI^2^)		*P* for interaction
	N	Q1	Q2	Q3	Q4	
**Gender**						0.2986
Male	2838	Ref.	0.72 (0.53, 0.99)	0.62 (0.45, 0.84)	0.64 (0.47, 0.86)	
Female	3044	Ref.	1.03 (0.75, 1.40)	0.93 (0.68, 1.27)	0.97 (0.71, 1.31)	
**Age**						0.9800
<65	3922	Ref.	0.87 (0.66, 1.14)	0.78(0.60, 1.01)	0.79 (0.61, 1.01)	
≧65	1960	Ref.	0.91 (0.63, 1.33)	0.77. (0.52, 1.13)	0.76 (0.50, 1.14)	
**Smoking**						0.3792
Smoker	2726	Ref.	0.99(0.71,1.38)	0.82(0.59,1.14)	0.73(0.53,1.00)	
Non-smoker	3156	Ref.	0.75(0.56,1.02)	0.72(0.53,0.96)	0.82(0.61,1.11)	
**Diabetes**						0.4439
Yes	997	Ref.	1.19(0.63, 2.27)	0.74 (0.39, 1.40)	0.70 (0.39, 1.26)	
No	4885	Ref.	0.81 (0.64, 1.02)	0.75 (0.59, 0.94)	0.79 (0.61, 1.00)	
**Hypertension**						0.3020
Yes	2840	Ref.	0.97 (0.64, 1.34)	0.83 (0.60, 1.15)	0.70 (0.51, 0.95)	
No	3042	Ref.	0.76 (0.57, 1.02)	0.71(0.53, 0.96)	0.85 (0.60, 1.14)	
**BMI**						0.8616
Noma weight	1437	Ref.	0.93 (0.60, 1.45)	0.76 (0.49, 1.16)	0.98 (0.62, 1.54)	
Overweight	2081	Ref.	0.86 (0.60, 1.23)	0.83 (0.58, 1.19)	0.83 (0.58, 1.18)	
Obese	2326	Ref.	0.79 (0.54, 1.15)	0.62 (0.43, 0.90)	0.65 (0.46, 0.91)	

^1^OR: odds ratio

^2^ 95%CI: 95% confidence interval

Adjusted for age, gender, race, education level, family to ratio of family income to poverty, BMI, smoking status, alcoholic status, hypertension, diabetes, CKD, cardiovascular disease, physical activity time, total energy intake except the subgroup variable

### Smooth curve fitting and inflection point detection

**Fig. 2:**
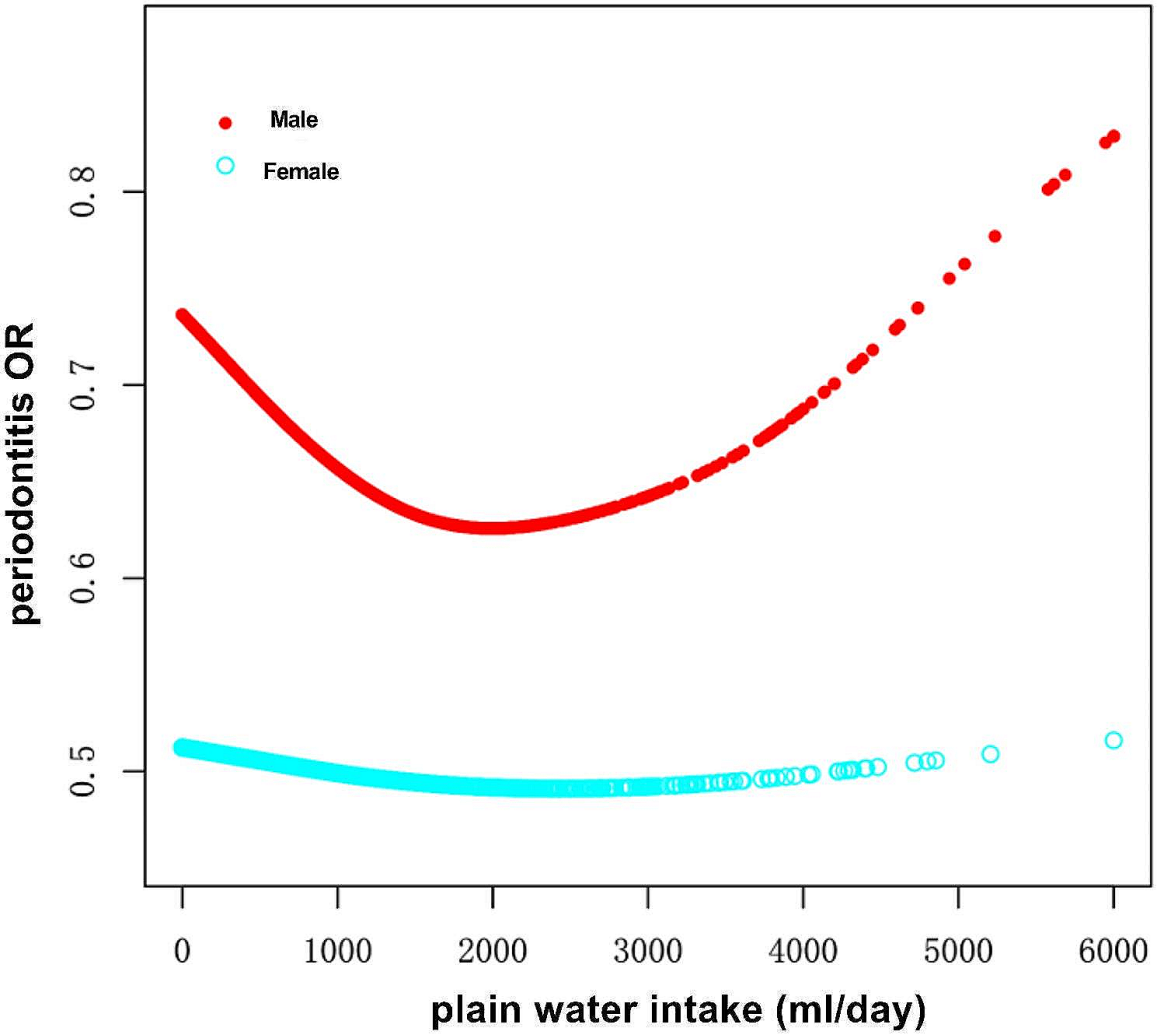
Smooth curve fitting stratified by gender

Figure 2 illustrates the smoothed curve fits stratified by gender. The curve for male participants follows a U-shape, while for women, the relationship approximates a straight line. Two-piecewise linear regression model analysis showed that for male participants, the inflection point of plain water intake was approximately 1200 ml/day. For males, the risk of periodontitis decreased by 24% with an increase of one unit of plain water intake (every 500 ml) when the intake was under 1200 ml/day (OR = 0.76, 95%CI 0.66–0.87, *p* < 0.001). When plain water intake was above 1200 ml/day, the increase of every 500 ml, the risk of periodontitis increased non-significantly (OR = 1.08;95%CI 0.99–1.18, *p* > 0.05). *p* < 0.001 in the log-likelihood ratio test.

## Discussion

In this large cross-sectional study based on the NHANES 2009–2014 cycles, we observed that participants in higher plain water intake quartile groups are associated with a lower incident of periodontitis. Subgroup analysis and interaction tests indicated that this trend was consistent in different population settings and was more pronounced in participants at a higher risk of periodontitis, namely males, elderly, smokers and individuals with hypertension and diabetes or obesity. Smooth curve fits stratified by gender showed that 1200 ml/day plain water intake was associated with the lowest periodontitis risk in males. Our findings suggested that adequate plain water intake is an independent protective factor for periodontitis.

Numerous studies have demonstrated the significant impact of diet on periodontal health. The intake of micronutrients, such as vitamins C [12], D [13, 14], E [15], and minerals such as calcium [20] and phosphorus [21], has been reported to be associated with the risk of periodontitis. In addition, some studies have also investigated the impact of macronutrient intake on the risk of periodontitis. For instance, high intake of carbohydrates [22] or saturated fat [23] is associated with a higher risk of periodontitis while fiber intake is inversely associated with periodontitis [24] and severe protein malnutrition leads to tooth loss [25].

Water is one of the most essential macronutrients, and adequate water intake is crucial for overall health. The recommended total fluid intake (including all fluid from drinking and food) is 3700 ml/day for men and 2500 ml/day for women. However, many individuals fail to meet this standard [26]. Generally, men and elderly populations are more likely to develop dehydration [26, 27]. The source of water intake also affects periodontal health. Soft drink [28] and alcoholic beverage consumption [29] were found to be associated with increased periodontitis risks. A few studies reported the association between coffee consumption and periodontal diseases [30–32], however a recent systematic review and meta-analysis by Yeonjae Rhee et al. concluded that no relationship between coffee consumption and periodontitis [33]. In contrast, green tea consumption was found to lower the risk of periodontitis [34]. Compared to other beverages, plain water is the most common, cheap, and easily gained water source. However, no study before has focused on whether plain water intake is associated with periodontitis. In our study, participants who drink more plain water have a significantly lower risk of periodontitis. In a recent study by David M Wright et al. [5], the association between certain dietary patterns and periodontitis was investigated, and their results showed one pattern which is rich in salad, fruit, and vegetables and with plain water or tea to drink, was associated with lower attachment loss. Together, it can be suggested that individuals at high risk of periodontitis should drink enough plain water to reduce the risk of developing the disease.

Interestingly, we found that there was no association between total water intake (water intake include moisture in food, plain water and other beverage) and the incidence of periodontitis (data not shown). This finding suggests that the association between plain water intake and periodontitis might not be directly related to systemic hydration status. This is similar to the findings of a study that investigated the relationship between water intake and the incidence of chronic kidney disease (CKD), where the intake of plain water, rather than total water intake, was found to be associated with CKD [35]. Therefore, it is important to focus on the intake of plain water.

We have proposed several potential mechanisms to explain the link between plain water consumption and the incidence of periodontitis. Firstly, individuals who consume higher amounts of plain water often consume fewer sugar-sweetened beverages, such as soda, sweet coffee, or other sugary drinks [36]. These beverages containing sugar, acid, and caffeine have been shown to have negative effects on periodontal health [28, 31]. Therefore, reducing their consumption through increased plain water intake may contribute to better periodontal health. Secondly, higher plain water intake has been associated with a more favorable metabolic profile in populations [7, 37–39]. Metabolic conditions, such as obesity and diabetes, can significantly impact periodontal health [40]. Therefore, increased plain water consumption may indirectly improve periodontal health through its positive effects on metabolic factors. Thirdly, individuals with low plain water intake have shown differences in gut microbiota compared to those with high water intake [41]. The gut microbiota has been implicated in influencing alveolar bone metabolism through the gut-bone axis [42, 43]. Therefore, changes in gut microbiota resulting from increased plain water intake may also play a role in the observed association with periodontitis. Additionally, it is worth noting that populations with higher education levels and better economic status tend to consume more plain water. These individuals may also have better oral hygiene habits, which can positively impact periodontal health. However, it is important to acknowledge that these explanations are speculative, and further studies providing direct scientific evidence are necessary to fully understand the relationship between plain water intake and periodontitis. Moreover, exploring the complex interactions between water consumption, socioeconomic factors, and periodontal health outcomes is crucial for a comprehensive understanding of this topic.

In our stratified study, in some of the subgroups, the association did not reach statically significance, which may be attribute to a reduced sample size in each subgroup. Notably, we found that the association between water intake and periodontitis differs between males and females. Interestingly, a study by Xing Wang et al. found a negative relationship between plain water intake and nonalcoholic fatty liver disease risk in males but not in females [44]. Likewise, plain water intake was associated with lower glycated Hb (HbA1c) in men but not in women [38]. We speculated that such difference between gender is possibly due to the fact that, overall, males require more water and are more susceptible to dehydration [26]. Alternatively, this difference may be related to sex hormone factors. Further research is needed to explain this discrepancy. Our study revealed a U-shaped relationship between plain water intake and the risk of periodontitis in men, suggesting that the lowest risk was associated with a plain water intake of 1200mL. This finding is consistent with a separate study on chronic kidney disease, which also found a U-shaped relationship and indicated that individuals consuming 1100–1500 mL of plain water had the lowest risks of CKD [35]. Based on our results, a plain water intake of 1200 mL may be an appropriate amount for men. However, it is important to note that our findings do not definitively prove harm from consuming more than 1200 mL due to limitations in statistical significance and sample size. Further research is required to better understand the underlying mechanisms behind this U-shaped relationship.

The strength of this study lies in the use of nationally representative NHANES data, providing a large sample size and enhancing the credibility of the conclusions drawn. However, it’s important to note that, as a descriptive study, our research can’t establish a causal relationship between water intake and periodontitis risk. The data on water intake, obtained through 24-hour dietary recall interviews, may be subject to recall bias and might not accurately represent participants’ long-term usual water intake. Furthermore, not all potential covariates were accounted for, and the generalizability of these findings to the young population remains uncertain. Future studies incorporating larger cohorts of young participants, RCTs, and laboratory research are warranted to elucidate this association and explore the underlying pathological mechanisms.

## Conclusion

Our study demonstrated that plain water intake was associated with periodontitis independently. Our study suggests that in the prevention and treatment of periodontitis in individuals over 45 years of age, especially for men, dietary recommendations should emphasize the role of adequate plain water intake, as it is a simple, inexpensive, and easily modifiable factor.

## Data Availability

The datasets generated and/or analyzed for this current study are not publicly available to protect participant confidentiality and are considered restricted meta data for administrative purposes and quality control. The National Center for Health Statistics may consider allowing limited access to some restricted data. Information to request access is available here: https://www.cdc.gov/rdc/. None -restricted data can be downloaded from the NHANES database (https://www.cdc.gov/nchs/nhanes/index.htm). The data used in the present study are available from the corresponding author on reasonable request.

## Abbreviations

95%CI: 95% confidence interval
AL: Attachment loss
BMI: Body Mass Index
CDC/AAP: Centers for Disease Control and American Academy Periodontology
CKD: Chronic kidney disease
NHANES: National Health and Nutrition Examination Survey
OR: Odds ratio
PD: Probing depth
